# Judicial Insanity—Trial of Nottidge *v.* Ripley

**Published:** 1849-10-01

**Authors:** 


### JUDICIAL INSANITY.

### TRIAL OF NOTTIDGE V. RIPLEY.

*Sittings at Nisi Prius, in Middlesex, after Trinity Term, before the* Chief Baron *and a Special Jury. June* 23*rd*, 25*th*, 26*th*, 1849.

Mr. Cockburn and Mr. M. Smith conducted the case for the plaintiff,—Sir F. Thesiger, Mr. Crowder, and Mr. Bovill, for the defendants.

This was an action to recover compensation in damages for the incarceration of the plaintiff in a lunatic asylum, under the pretence that she was of unsound mind at a time when, in truth, the plaintiff, a maiden lady, was perfectly sane.

The pleas put upon the record in answer to the allegations set forth in the declaration, pleaded, first, " Not guilty," and then that the plaintiff was of unsound mind, and that it was unsafe for herself and for others that she should be at large.

Mr. Cockburn opened the case. The plaintiff, Miss Nottidge, was a maiden lady of middle age, possessing 6000*l*. in the funds. Towards the end of 1846, she went to reside at Charlinch, in Somersetshire, to enable her to enjoy the society of her three sisters, who were married to gentlemen, named Price, Thomas, and Cobb, members of a certain sect or fraternity settled in that locality, and living all together in an establishment called the " Agapemone." Miss Nottidge had lodgings in a cottage close by, and remained there until the 12th of November, 1846, when she was seized by her brother-in-law (the defendant Ripley), assisted by others. and forcibly conveyed to Dr. Stillwell's lunatic asylum at Hillingdon, Here the plaintiff was kept until January, 1848, when she was liberated by order of the Commissioners in Lunacy. She now sought compensation for her long incarceration.

A witness, George Waterman, proved the abduction, and identified the defendant Ripley.

Joan Waterman—kept the lodgings occupied by the plaintiff; witnessed the abduction; considered plaintiff " a nice quiet lady, not dangerous to herself or others."

Eliza Waterman.—Waited on plaintiff. She was quiet and gentle in her conduct, and not at all like a mad woman.

Lewis Price, brother-in-law to plaintiff, dwells with his wife in the Agapemone, which he describes as a "private family dwelling together and entertaining peculiar religious views—in which views the plaintiff concurred." Had never seen any unsoundness of mind in plaintiff, but always found her gentle, quiet, and ladylike. Was mainly instrumental in procuring her liberation by the Commissioners in Lunacy. (This witness then gave some details respecting the Agapemone—the peculiar religious views and customs of its inmates.) Had received 6000*l.* with the sister of plaintiff, and Messrs. Thomas and Cobb had done the same. There were no marriage settlements made. Plaintiff had been at the "Agapemone" ever since her return from the asylum. When she came back, she made over all her property to Mr. Prince, the chief of the community, to protect it from any other persons, and from the defendants. Her money is in the bank, in Mr. Prince's name. Plaintiff never played at "hockey," but sometimes rode out in the carriage and four.

William Cobb, brother-in-law to plaintiff.—Had 6000*l.* with his wife. Has resided in the Agapemone nearly seven years. Considers Miss Nottidge perfectly free from madness. She had told him that she did not look upon Mr. Prince as God, that she did not believe that gentlemen to be God; had never heard her say that she looked up to Mr. Prince as God, or anything of the kind. Plaintiff was in a perfectly sound state of mind.

Mrs. Prince.—Has known the plaintiff for six or seven years. She was always of a calm, gentle, ladylike frame of mind. Since her return from the asylum, she has been quite the same. We have recreations at the Agapemone on Sundays, as well as on other days. We believe in the Trinity, but do not pray. We abjure all prayer, because we consider that the day of prayer is past, that the day of grace is past, and that the day of judgment has arrived.

Mrs. Harriet Price.—Is sister to plaintiff: considers her steady, calm, tranquil, and lady-like. Never saw anything to the contrary. Since plaintiff returned to the Agapemone from the asylum, her mind is just as calm as it was before—as it has always been.

Mrs. Cobb.—Is sister to plaintiff. She is perfectly sound in her mind, very gentle, and particularly mild. She has never exhibited the least symptom of insanity. Mr. Ripley, my brother-in law, and my brother, the clergyman, the defendants, have always been very kind to us all. Before we were married at Swansea, my mother came down to prevent our marriages, but I and my sisters considered it to be the will of God that we should marry these gentlemen, and therefore we refused to accompany my mother back. After our marriage, my mother came to Weymouth to search for the plaintiff, but I would not give her any information as to where she was, because I knew it was her wish that her mother should not know. This was about June, 1846. I am of opinion that the day of grace has passed, and the day of judgment arrived. Plaintiff entertains the same notions, and I consider her to be of sound mind. She is as perfectly sane as myself.

G. V. Thomas, brother-in-law to plaintiff.—Remembers Miss Nottidge being taken away from Charlinch. Her mind was perfectly sane and proper in every respect. She has never exhibited the slightest symptom of unsoundness of mind, nor any delusion. Went to the asylum to fetch her away, and did not observe any difference in her mind. She has been in the Agapemone ever since, perfectly sound in mind and manner.

Arthur Mayburn.—Is a surgeon; lives in the Agapemone, and practises among the inmates. Knows plaintiff; she is gentle in temper and of perfectly sound mind. Has never observed any sign of a delusion.

John Williams, gentleman, explained at length the peculiar religious tenets of the sect.

Sir F. Thesiger then addressed the jury on behalf of the defendants, and produced the following evidence :—

Mrs. Nottidge, mother to the plaintiff.—(After detailing the circumstances of the desertion and marriage of her other three daughters, and the absconding of the plaintiff, continued.)—Plaintiff came to see me in London, accompanied by her sisters. I put down on paper what she said. I asked about Mr. Prince; she said, "I know no such person. God now dwells only at Charlinch, in the flesh of him I once knew as Mr. Prince. God, who made me and all the world, is now manifest in him I once called Mr. Prince. He has entered his tabernacle of flesh among men, and I have seen God face to face. He will deliver me wherever I am taken." After that, witness sent for her brother and the medical men.

Mr. John Pepys, uncle to the plaintiff.—Is of opinion that her mind was under a strong delusion. Talked to her for some time, but obtained no answer beyond "That it was the Lord's will that she should live down at Charlinch." Advised that a medical man should be sent for, and that she should be watched.

Thomas Morton, F.R.C.S.—Saw Miss Nottidge on Nov. 11th, 1846, and then certified that "she had of late estranged herself from her mother's house to follow a person of the name of Prince, whom she believed to be Almighty God, and herself immortal." When asked whether she did not believe that Mr. Prince *was* Almighty God she refused to answer, but when asked whether she did not believe that Mr. Prince *was not* Almighty God, she said that she did not believe that he *was not* the Almighty.

Dr. Rowland.—Plaintiff told him that she was a disciple of Mr. Prince's, and that she could not deny having the belief that Mr. Prince was Almighty God. Was perfectly satisfied at the time of her unsoundness of mind.

Dr. Stillwell, head of the Moorcroft House Asylum at Hillingdon.—Received plaintiff at his establishment, and did all he could to be of service to her. She told him that "Mr. Prince was God manifest in the flesh; that the day of grace had passed, and the day of judgment had come. She also said that Mr. Prince had rendered her immortal—that she should not die—that she should not be buried in a coffin as other persons were, but taken up to heaven in the twinkling of an eye. She said that she had ceased to pray, and only sang praises to God." These she sang as she walked about the room, but never made use of any intelligent words. She entertained great antipathies to her mother and friends. She repeated the above remarks relative to Mr. Prince on May 9th, only a week before her discharge.

Mr. Lutwidge, secretary to the Lunacy Commissioners, showed from the minutes that Dr. Stillwell's asylum had been visited by the commissioners as many as eight times while plaintiff was an inmate, and that her case had been considered at six of those visits.

Dr. Turner proved visiting the asylum in Dec. 1846, and seeing plaintiff. She would not converse, but she said, "I believe in the same God as you do," and would not answer when I asked her whether she considered Mr. Prince to be God Almighty. The result of the interview was, that the commissioners had no doubt whatever of her unsoundness of mind. However, she was subsequently discharged on the ground of her declining health.

Mr. Mylne, one of the Commissioners in Lunacy, and a member of the bar, who said that in the course of his duty as one of the Lunacy Commissioners, he saw the present plaintiff, Miss Louisa Nottidge, on two occasions, whilst she was a patient in the establishment of Dr. Stillwell. The case of that lady had previously been the subject of much inquiry and discussion amongst the commissioners at the various meetings. He saw the lady, in the first instance, in the company of Dr. Pritchard, and on that occasion, from his examination of the lady, he was quite satisfied of her unsoundness of mind. Unfortunately, Dr. Pritchard had died since; but he could state that that lamented and able gentleman had entertained a much stronger opinion even than himself as to the plaintiff being a very fit object for confinement in the asylum.

The Lord Chief Baron.—Mr. Mylne, was this lady in such a state of mind as to be dangerous to herself or to others?

Mr. Mylne.—Not so as I was aware of; not so far as I knew.

The Lord Chief Baron.—If she were not so, then, how was it that you kept her in this asylum for seventeen months?

Mr. Mylne.—My lord, it was no part of my duty to keep her there. I was only to liberate her if I saw good and sufficient reason for adopting that course.

The Lord Chief Baron.—It is my opinion that you ought to liberate every person who is not dangerous to himself or to others. If the notion has got abroad that any person may be confined in a lunatic asylum or a madhouse who has any absurd or even mad opinion upon any religious subject, and is safe and harmless upon every other topic, I altogether and entirely differ with such an opinion; and I desire to impress that opinion with as much force as I can in the hearing of one of the commissioners.

Mr. Mylne's examination continued.—The second time he saw this lady, she had declined and refused to enter into any private conversation with the commissioners, and therefore, in reference to that interview, they had not made any entry in the book. He was himself called to the bar in the year 1827, and had been appointed one of the Commissioners in Lunacy in 1832. It had been the unanimous opinion of the commissioners that Miss Nottidge was of unsound mind up to May, 1848.

The Lord Chief Baron.—You say of unsound mind, Mr. Mylne. Had she any unsoundness of mind upon any other subject under heaven except as to entertaining these peculiar religious notions?

Mr. Mylne.—Miss Nottidge did not exhibit any symptoms of insanity upon any other subject. It was understood by the commissioners, in the month of May, that the health of the patient was suffering from the continued confinement, and they had, therefore, felt themselves justified in directing that she should be set at liberty. There were several persons under the care of Dr. Stillwell in his asylum, in respect of whom no commission *de lunatico inquirendo* had issued. There were about 500 patients who were in confinement under the warrants of the commissioners, and there were about 15,000 who were in confinement in asylums, in respect of whom there had been no commission of inquiry; 9000 of that number, however, were in the pauper asylums.

The next witness, Mr. Proctor, said, he had been a Commissioner in Lunacy as many as seventeen years. He had seen this lady on the 1st of May, 1848. Monomaniacs were frequently under delusions in reference to matters of religion, and others in respect of other subjects. One of the results of that visit on the 1st of May was, that the patient was liberated. That which had passed upon the 1st of May at the meeting of the commissioners was reduced to writing, and put into the form of a report, which had been drawn up by himself. The witness had been of opinion that in

May, 1848, the period at which Miss Nottidge had been liberated, her delusions on religious subjects still continued. Indeed, the circumstance of her having, since her release, transferred all her property over to Mr. Prince, the individual who, all through the proceedings, had appeared to be the main object of her delusions, had subsequently satisfied him that not only was she of unsound mind, but insane. The commissioners did not think it advisable, where the disease appeared likely to be temporary only, that a Commission of Lunacy should be applied for.

Mr. Stedman was the medical attendant upon the family of the defendant Ripley. He had been called to see this lady, but he had declined to act in consequence of his being connected with the family, and he therefore suggested that other medical advice should be called in.

Mr. Cockburn replied.

The Lord Chief Baron proceeded to sum up the evidence. His Lordship said, that the jury must find a verdict for the plaintiff upon the plea of "not guilty," because the evidence had clearly proved that the defendants had been guilty of a portion of the acts which had been charged against them; for it was proved that they had gone down to Charlinch and dragged the plaintiff from the peaceful and quiet home which she had selected; and then as to the next plea—the plea of justification—namely, that the plaintiff was a lunatic, and not capable of taking care of herself, and was in such a state of mind as to be likely to injure herself and other persons, and that it was in consequence of her being in that state that they had had her put in a place of safety—that plea was not made out. Now, he was bound to say, that the law had been correctly laid down by the learned counsel who had just sat down—namely, that if the jury considered, upon the evidence, that the plaintiff was not in such a state as to be dangerous to herself and others, then, that the plea to that effect not having been made out, the verdict ought to be for the plaintiff upon that issue also. The evidence had clearly shown, as applicable to this branch of the case, that the conduct of the plaintiff had been perfectly consistent, even in respect of her delusions. The lady entertained a strong opinion on a religious subject, and to that opinion, whether right or wrong, she had adhered. Then it appeared to him that the defendants were not in any way justified in having adopted the course they had taken, unless the jury should think that the plaintiff was of unsound mind, and dangerous to herself and others. If she were not so, then the defendants had no right to go down and drag her away, as they had done, from her home, for Charlinch was the home she had chosen, and then to cause her to be placed in an asylum. If, then, the jury should be of opinion that the plaintiff was not in that state of danger, it was clear their verdict upon the second plea must be for the plaintiff also. The question, however, was entirely for the jury, and they must exercise their own judgment in the matter. If they should in the end determine upon finding for the plaintiff, then they would have to consider what amount of damages they would award to a lady who, although perfectly sane, unless it were upon the subject of religion, had been incarcerated in a lunatic asylum for the space of seventeen months. There could be no doubt, if this lady was not insane and dangerous, that a most unjustifiable outrage had been committed upon her by the defendants in their own persons—a most unjustifiable outrage had been committed upon a lady who, it was admitted, was upon all other subjects perfectly sane. But the defendants had said that they had taken this step for the purpose of rescuing a beloved relative from the pernicious examples of a class of persons who entertained certain peculiar notions upon religion. His own idea of toleration was, that all those who entertained with sincerity any peculiar doctrine, however absurd that doctrine might appear to others to be, ought to be allowed to enjoy that opinion without interference, so long as the principles and the acts they adopted, were not forced offensively, or contrary to law, upon the public notice, or against the public morals. If such persons sincerely entertained these doctrines, then they were, in his opinion, as much entitled to be treated with respect as any other religious sect. But whilst this was so, the majority of persons in this country might look with horror and disgust at the views which these people had adopted. This was only one of the many bursts of strange fancies, with regard to religion, with which the country had been visited upon various occasions. There was one point in this transaction upon which he felt called upon to offer a remark. It was, that whilst three of these sisters had been married to three members of this fraternity, not one of them had had the precaution of having a settlement drawn. It would have been not merely wise, but nothing more than right, that settlements should have been made to provide a maintenance for the wives, in the event of anything happening to the husbands. It would have been well if these gentlemen had had this done, for much cause of suspicion, of imputed motives in uniting themselves to ladies much older than themselves, would thereby have been removed. He very much doubted whether, if in this case the plaintiff had been a man, or living under the protection of a husband, the defendants would have dared to have taken the step they had. When consulted by the mother, they ought to have refused to have taken that step, until a medical examination, or an inquiry by commission, had been made. But they had not done so, and therefore they had made themselves liable to such a verdict as the jury might think fit to impose upon them.

The jury retired for an hour, and then came back with a verdict for the plaintiff, damages 50*l.*, and said, they begged to give their opinion that the defendants had not been actuated by any mercenary or unworthy motives in the steps they had taken.

The damages were laid at 1,000*l.* in the declaration.

---

